# Long Cycle Life for Rechargeable Lithium Battery using Organic Small Molecule Dihydrodibenzo[c,h][2,6]naphthyridine‐5,11‐dione as a Cathode after Isoindigo Pigment Isomerization

**DOI:** 10.1002/advs.202307134

**Published:** 2023-11-30

**Authors:** Mingcong Yang, Wei Hu, Jun Li, Tao Chen, Shiqiang Zhao, Xi'an Chen, Shun Wang, Huile Jin

**Affiliations:** ^1^ Key Lab of Advanced Energy Storage and Conversion Zhejiang Province Key Lab of Leather Engineering College of Chemistry and Materials Engineering Wenzhou University Wenzhou Zhejiang 325035 China; ^2^ Zhejiang Engineering Research Center for Electrochemical Energy Materials and Devices Institute of New Materials and Industrial Technologies Wenzhou University Wenzhou Zhejiang 325035 China; ^3^ Department of Materials Science and Engineering School of Chemistry and Materials Science University of Science and Technology of China Hefei Anhui Province 230026 China

**Keywords:** high cycling stability, high‐capacity retention, lithium battery, organic cathode, organic small molecular cathode

## Abstract

Sustainability and adaptability in structural design of the organic cathodes present promises for applications in alkali metal ion batteries. Nevertheless, a formidable challenge lies in their high solubility in organic electrolytes, particularly for small molecular materials, impeding cycling stability and high capacity. This study focuses on the design and synthesis of organic small molecules, the isomers of (E)‐5,5′‐difluoro‐[3,3′‐biindolinylidene]‐2,2′‐dione (EFID) and 3,9‐difluoro‐6,12‐dihydrodibenzo [c, h][2,6]naphthyridine‐5,11‐dione (FBND). While EFID, characterized by a less π‐conjugated structure, exhibits subpar cycling stability in lithium‐ion batteries (LIBs), intriguingly, another isomer, FBND, demonstrates exceptional capacity and cycling stability in LIBs. FBND delivers a remarkable capacity of 175 mAh g^−1^ at a current density of 0.05 A g^−1^ and maintains excellent cycling stability over 2000 cycles, retaining 90% of its initial capacity. Furthermore, an in‐depth examination of redox reactions and storage mechanisms of FBND are conducted. The potential of FBND is also explored as an anode in lithium‐ion batteries (LIBs) and as a cathode in sodium‐ion batteries (SIBs). The FBND framework, featuring extended π‐conjugated molecules with an imide structure compared to EFID, proves to be an excellent material template to develop advanced organic small molecular cathode materials for sustainable batteries.

## Graphical abstract

The novel organic cathode, 3,9‐difluoro‐6,12‐dihydrodibenzo [c, h][2,6]naphthyridine‐5,11‐dione (FBND), is synthesized in success in this paper, which showed enhanced stability with 90% capacity retention over 2000 cycles for energy storage after isomerization of isoindigo pigment (EFID), indicating FBND framework boosts cycling stability. Meanwhile, it showed dual utility in lithium‐ and sodium‐ion batteries. Besides, density functional theory calculations is adopted to confirm mechanism of charge–discharge process.

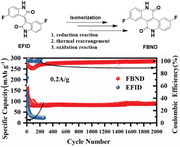

## 1 Introduction

Over the past three decades, lithium‐ion batteries (LIBs) utilizing intercalated cathodes have experienced remarkable success in powering various applications, including portable electronics, grid‐scale energy storage, and electric vehicles. Currently, cathode materials can be categorized into inorganic and organic materials. Inorganic cathodes, while effective, suffer from limited capacity with compatibility restricted to single metal ions, escalating costs due to energy‐intensive and environmentally harmful synthesis and disposal processes, limiting their further adoption in the expanding energy storage market.^[^ ^1^ ^]^ In contrast, organic materials offer designated theoretical capacity, cost‐effectiveness, eco‐friendliness, compatibility with multiple metal ions, and tunability of electrochemical performance, making them a promising solution for green electrochemical energy storage.^[^ ^2^, ^3^, ^4^, ^5^ ^]^

However, a significant challenge confronting organic cathodes is the dissolution of organic materials in organic electrolytes during the charge/discharge cycling process. This phenomenon leads to capacity fading, as dissolved original materials or redox products disperse from the conductive electrode network into the electrolyte or pass through the separator to the counter electrode, resulting in adverse effects on electrochemical performance, particularly in terms of cycling stability. To address this issue, several strategies have been explored, including enlarging the π‐conjugated system,^[^ ^6^, ^7^, ^8^, ^9^ ^]^ polymerization,^[^ ^10^, ^11^, ^12^, ^13^, ^14^ ^]^ forming organic salts,^[^ ^15^ ^]^ and incorporating organic materials into porous carbon composites.^[^ ^16^, ^17^, ^18^, ^19^, ^20^ ^]^ Another approach involves improving the electrolyte and separator, such as using aqueous electrolytes,^[^ ^21^ ^]^ high‐concentration electrolytes,^[^ ^22^ ^]^ or solid electrolytes,^[^ ^23^, ^24^, ^25^ ^]^ which have demonstrated excellent cycling performance due to the similarity‐intermiscibility theory, where saturated solvents reduce solvent activity and inhibit the dissolution of active materials or their redox products into the electrolyte.

Generally, polymerization of small molecules has been an efficient strategy to inhibit dissolution and enhance cycling stability,^[^ ^11^ ^]^ but it often results in poor access of cations and electrons to the redox active groups, leading to low utilization efficiency of theoretical capacity and costly synthesis. A typical quinone small molecule organic material, C_6_O_6_,^[^ ^26^ ^]^ shows the highest theoretical capacity of 957 mAh g^−1^ for organic cathode, while a typical polymer cathode with theoretical capacity in range of 150–300 mAh g^−1^, i.e., polyaniline with pernigraniline base.^[^ ^27^ ^]^ Notably, small organic molecules exhibit higher potential for capacity but are hindered by their solubility in aprotic electrolytes and inherently low electronic conductivity. Balancing high capacity and cycling stability in small molecule organic cathodes poses a formidable challenge. For instance, certain quinones, like cyclohexanehexone (C_6_O_6_^[^ ^24^, ^26^ ^]^) and Calix[4]quinone (C_4_Q^[^ ^17^ ^]^), have demonstrated high initial capacity in common electrolytes but degraded to a fraction of their initial capacity after only a few cycles in LIBs. Similarly, some anhydrides, like Perylene‐3,4,9,10‐tetracarboxylic dianhydride (PTCDA^[^ ^28^ ^]^) and 1,4,5,8‐Naphthalenetetracarboxylic dianhydride (NTCDA^[^ ^29^ ^]^), showed exceptionally high initial capacity but suffered severe capacity fading in LIBs. These examples underscore the difficulty in achieving both high capacity and cycling stability in organic materials.

Recently, the incorporation of hydro bond strategies in certain organic compounds, like 2, 7‐ diamino‐4, 5, 9, 10‐tetraone (PTO‐NH_2_),^[^ ^30^ ^]^ has shown promising results in improving cycling stability. However, the use of imide structures, which also possess hydro bond interactions, has shown mixed results. For example, 3,4,9,10‐Perylene‐bis(dicarboximide) (PTCDI^[^ ^31^ ^]^) with an imide structure and higher π‐conjugated system demonstrated high reversible capacity in sodium‐ion batteries (SIBs), while 1,4,5,8‐naphthalene‐bis(dicarboximide) bis(dicarboximide) (NTCDI^[^ ^32^ ^]^) with an imide structure showed poor cycling stability in LIBs. Additionally, organic pigment 5,5′‐indigodisulfonic acid sodium salt^[^ ^33^, ^34^, ^35^ ^]^ maintained stable capacity in both SIBs and LIBs, suggesting multiple metal ion compatibility as a cathode material.

In light of these challenges and successes, the isomers of indigo, specifically isoindigo derivatives, which are typically applied as acceptors of copolymers for electrochemical transistors^[^ ^36^ ^]^ and solar cells^[^ ^37^ ^]^ due to its electron‐deficient properties, have garnered attention as potential candidates for cathodes in LIBs and SIBs. Incorporating polarized groups, such as ‐CN, ‐OH, and ‐X (F, Cl, Br, I), into molecules has been proposed to reduce solubility and improve cycling stability.^[^ ^9^, ^38^ ^]^ Hence, fluorine substitution of isoindigo may hold promise for enhancing cycling stability through the hydro bond of imide group. In this study, we synthesized (E)−5,5′‐difluoro‐[3,3′‐biindolinylidene]−2,2′‐dione (EFID) and investigated its performance as a cathode material in LIBs. Surprisingly, the isomer 3,9‐difluoro‐6,12‐dihydrodibenzo[c,h][2,6]naphthyridine‐5,11‐dione (FBND) after isomerization of EFID exhibited high capacity and excellent cycling stability in LIBs. FBND delivered a capacity of 175 mAh g^−1^ at a current density of 0.05 A g^−1^ and maintained good cycling stability over 2000 cycles with an impressive retention of 90%. Moreover, we explored FBND's potential as an anode material in LIBs and as a cathode material in SIBs.

This paper presents the first study of EFID and its isomer FBND as energy storage materials, along with their corresponding storage mechanisms. The findings provide a new design strategy for organic cathodes based on small molecular materials with high cycling stability and rich organic small molecule electrode potential.

## 2 Results and Discussion

### 2.1 Synthesis and Structure Characterization

**Figure 1:**
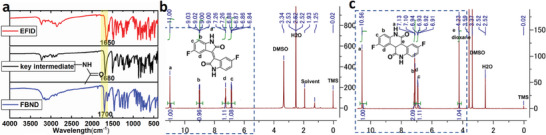
Structure characterization of EFID and FBND: a) FTIR spectra involving EFID, intermediate product, and FBND; b,c) H‐NMR spectra for EFID and intermediate product.

The EFID was synthesized using an aldol reaction with 5‐fluoroindoline‐2,3‐dione and 5‐fluoroindolin‐2‐one as readily available starting materials under acidic conditions using HCl in AcOH, resulting in a high yield of 95%. The confirmation of EFID's structure was carried out through H‐NMR spectroscopy, as shown in **Figure** **1**b. On the other hand, FBND was synthesized by isomerization of EFID through a two‐step process in high yield of 81% illustrated in Scheme S1. Due to its poor solubility in common solvents, FBND was characterized using only FTIR, as depicted in Figure 1a.

The isomerization process involves several sequential steps. First, the double bonds in the center of EFID were reduced to a single bond using Zn dust in THF solvent with CF3COOH. Subsequently, the compound underwent a ring‐expanding re‐lactamization reaction, leading to the formation of a key intermediate in yield of 82%. This key intermediate was characterized by FTIR and H‐NMR (Figure 1a,c) in accordance with Scheme S1. Finally, the key intermediate underwent an oxidation dehydrogenation reaction under conditions of oxygen and base, resulting in the formation of the final product, FBND in yield of ≈99%. Further details on these operations are presented in the synthesis section of the experimental part.

Various characterization methods were employed to confirm the structures of EFID, the key intermediate, and FBND. For EFID, the H‐NMR spectra displayed characteristic peaks at chemical shifts of 11.05, 9.0, 7.25, and 6.87 ppm, corresponding to N‐H and three types of Ar‐H positions (Figure 1b). Mass spectra further corroborated the successful synthesis of EFID, revealing a peak at 299.0625 (Figure S1a, Supporting Information). Similarly, for the key intermediate, the H‐NMR spectra showed characteristic peaks at chemical shifts of 10.57, 7.19‐7.18, 6.95, and 4.23 ppm, corresponding to N‐H, C‐H at position e, and three types of Ar‐H positions at b, d, and d, respectively (Figure 1c). Mass spectra confirmed the structure of the key intermediate, with a peak at 300.0 (Figure S1b, Supporting Information).

In the case of FBND, both the key intermediate and the final product were characterized using FTIR, represented by the black and blue lines in Figure 1a, respectively. The measured IR spectrum revealed a characteristic peak ≈1700 cm^−1^, attributed to the vibrational bending mode of the ─C═O structure in all three compounds. Since these compounds share a similar structural feature, they displayed analogous peaks in the IR spectrum. FBND was successfully obtained from the key intermediate according to Scheme S1, and a slight red shift in the vibrational wavelength of the ─C═O structure, from 1680 to 1700 cm^−1^, indicated a minor change in the structure, possibly due to an expansion of the conjugated system.

X‐ray diffraction (XRD) patterns were initially collected to investigate the crystal structure and phase information of the EFID and FBND materials. Figure S2 (Supporting Information) illustrates the highly crystalline nature of both EFID and FBND. The morphology of EFID and FBND was further examined using scanning electron microscopy (SEM) and transmission electron microscopy (TEM), as depicted in Figures S3 and S4 (Supporting Information), respectively. Both EFID and FBND exhibited a composition of a large quantity of one‐dimensional rods, with FBND preferring a squarer shape. The SEM and TEM images indicated that these rods had a length of approximately several microns. Additionally, energy‐dispersive X‐ray spectroscopy (EDS) mapping was performed for both EFID and FBND, confirming a uniform distribution of carbon (C), nitrogen (N), oxygen (O), and fluorine (F) elements, as they are isomers with the same elemental composition (Figures S3 and S4, Supporting Information).

To evaluate the solubility of EFID and FBND, solubility investigations were conducted by immersing equal amounts of powder in electrolyte. Notably, the electrolyte with EFID rapidly turned red, indicating the dissolution of the compound, while the electrolyte with FBND remained transparent over 14 days under the same conditions, as evidenced in photographs in Figure S5b and a (Supporting Information), respectively. UV–vis spectra of EFID and FBND in the electrolyte were also measured as shown in Figure S5 (Supporting Information). When EFID was immersed in the electrolyte, a strong UV‐vis signal was observed, indicating its high solubility. Conversely, FBND immersed in the electrolyte after 14 days exhibited minimal changes in its UV–vis signal, suggesting its low solubility. Furthermore, the elemental composition and distribution within FBND were examined after discharging to 1.2 V and after charging to 3.8 V, as verified by EDS mapping (Figure S6, Supporting Information), exhibiting a uniform distribution of C, N, O, and F. This observation indicates that the intermediate products of FBND formed during the discharge/charge process are stable, which is an important factor contributing to the long‐term cycling stability of the electrodes.

### 2.2 Electrochemical Performance of the Isomers (EFID and FBND)

**Figure 2:**
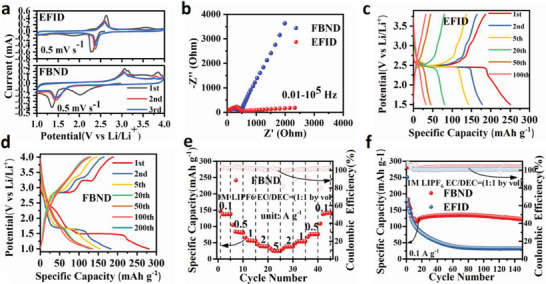
Electrochemical performance of EFID and FBND: a) Cyclic voltammetry (CV) curves at a scan rate of 0.5 mV s^−1^. b) Impedance plots of EFID and FBND before the self‐discharge test. c,d) Discharge‐charge curves of EFID and FBND at a current density of 0.1 A g^−1^. e) Rate performance of FBND at various current densities. f) Cycling performance of EFID and FBND at 0.1 A g^−1^.

The electrochemical behavior of EFID and FBND cathodes in lithium batteries was evaluated using coin cells with lithium foil as anode electrode, polypropylene membrane as the separator (Celgard 2500), and a 1.0 M LiPF_6_ in EC: DEC solution mixed with 1:1 volume ratio as the electrolyte. Cyclic voltammetry (CV) was performed over a voltage range of 1.0–4.0 V with a scan rate of 0.5 mV s^−1^, as depicted in **Figure** **2**a. Two distinctive pairs of oxidation/reduction peaks were observed in the CV for both EFID and FBND, stabilized at ≈2.4 and 2.6 V for EFID and ≈1.6 and 3.1 V for FBND, respectively. FBND also displayed a split oxidation peak which is similar to PTCDA^[^ ^14^ ^]^ at 3.4 V, possibly attributed to two kinds of the reduction product such as radical anion and dianion state.^[^ ^39^ ^]^ The isomers exhibited a pair of peaks, indicating a one‐step reversible enolization process (‐C = O → ‐(C‐O)‐M+) referred from some papers on imide compounds^[^ ^31^ ^]^ or carbonyl compounds,^[^ ^15^, ^26^, ^40^ ^]^ resulting in the same theoretical capacity of 179 mAh g^−1^ calculated by the Equation (3). However, the peak potential positions were notably different due to the change in the energy gap between the highest occupied molecular orbital (HOMO) and lowest unoccupied molecular orbital (LUMO) levels after the isomerization of EFID to FBND, as indicated by the apparent color change as **Figure** **5** and computational analysis by Gaussian 09 as Figure S7 (Supporting Information).

Electrochemical impedance spectroscopy (EIS) measurements in Figure 2b revealed that both EFID and FBND exhibited large interface resistance (≈480Ω) between the electrolyte and the electrode, indicating poor rate performance. Figure 2c,d show the initial discharge/charge capacity of ≈250/175 mAh g^−1^ for EFID and ≈275/175 mAh g^−1^ for FBND at 0.1 A g^−1^, with coulombic efficiencies of ≈70% and 67%, respectively. The subsequent curves after 20 activation cycles almost overlapped for FBND cathode with a specific capacity of 135 mAh g^−1^, and at a smaller current of 0.05 A g^−1^ (as shown in Figure S8, Supporting Information), it exhibited a capacity of 175 mAh g^−1^. In contrast, the EFID cathode quickly declined to ≈150 mAh g^−1^ within 5 cycles of 20 activation cycles and then gradually decayed to 50 mAh g^−1^ after 50 cycles, displaying a continuous capacity decay due to its higher solubility compared to FBND, as confirmed by solubility measurements in the electrolyte using UV–vis spectra (Figure S5, Supporting Information).

Regarding cycling stability, the FBND cathode demonstrated remarkable stability as shown in Figure 2f. When cycled at 0.1 A g^−1^, it achieved a specific capacity of 135 mAh g^−1^ from the 20th to the 100th cycle (after 20 activation cycles), with capacity retention of approximately 100% and a high retention of 77% of the initial charge cycled capacity, comparable to the commercial cathode material of LiFePO_4_.^[^ ^41^ ^]^ In contrast, the EFID cathode quickly decayed to 95 mAh g^−1^ with a retention of 54% of the initial charge cycled capacity after 20 activation cycles, and further decreased to 30 mAh g^−1^ with a retention of 17% after 100 cycles. This indicates that the isomerization of EFID into FBND improved the electrochemical performance by rendering the material insoluble and enhancing cycling stability.

Moreover, the rate capability of FBND was evaluated after 100 cycles at progressively increased current levels. Figure 2e demonstrates stable reversible capacities of 135, 85, 55, 45, and 28 mAh g^−1^ at current rates of 0.1, 0.5, 1, 2, and 5 A g^−1^ for FBND, respectively. Each current level exhibits symmetrically reversible capacities, indicating a consistent trend. However, at a very high current density of 5 A g^−1^, FBND cathode only presents a small reversible capacity of 28 mAh g^−1^, corresponding to only 21% of the capacity at 0.1 A g^−1^ (135 mAh g^−1^). This suggests that FBND's rate performance is poor due to its large interface resistance, as observed in the electrochemical impedance spectroscopy (EIS) measurements in Figure 2b, and the low ions diffusion coefficient ($D_{Li^{+}}$) in FBND, which will be discussed in the electrode kinetics section. The FBND's bad rate capacity is consistent with many other organic cathode materials reported previously,^[^ ^11^, ^12^, ^42^ ^]^ attributed to the low conductivity of organic materials.

It is evident that the isomerization of EFID to FBND did not help improve the rate capability of the material. On the contrary, the increasing energy gap after isomerization of EFID to FBND resulted in reduced conductivity of the active material. However, despite this reduction in conductivity, FBND exhibited better capacity and electrochemical stability compared to the EFID cathode, indicating that the isomerization of EFID into FBND resulted in improved electrochemical performance.

The effect of the electrolyte on the electrochemical performance of the organic cathodes, EFID and FBND, was investigated using 1 M LiTFSI/DOL‐DME (LiTFSI dissolved in a mixture of 1,3‐dioxolane (DOL) and 1,2‐dimethoxyethane (DME) in a volume ratio of 1:1) as a replacement for the commercial electrolyte of 1 M LiPF_6_/EC‐DEC (volume ratio of 1:1). The LiTFSI/DOL‐DME electrolyte is commonly used in organic cathodes^[^ ^19^ ^]^ or lithium‐sulfur batteries^[^ ^43^ ^]^ to form a stable solid electrolyte interface (SEI) ^[^ ^44^, ^45^ ^]^ and prevent active material dissolution into the electrolyte.

**Figure 3:**
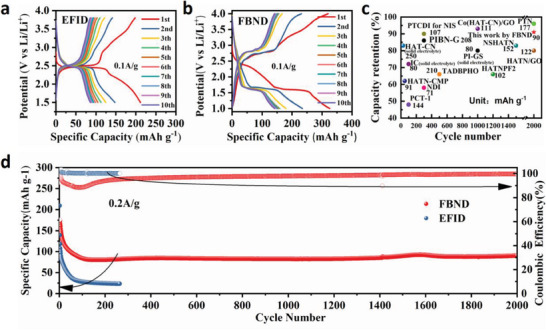
Electrochemical performance of EFID and FBND with LiTFSI/DOL‐DME as electrolyte: a,b) Discharge‐charge curves of EFID and FBND at the current density of 0.1 A g^−1^; c) Capacity retention comparison of this work with reported organic cathode material for stable cycling; d) Long cycling performance of EFID and FBND at 0.2 A g^−1^ for stability measurement.

The electrochemical properties of EFID and FBND cathodes were measured in coin cells with lithium as the anode. **Figure** **3**a,b show the capacity‐voltage profiles of EFID and FBND at a current density of 0.1 A g^−1^. The initial discharge/charge capacities of EFID and FBND were found to be 220/200 and 320/310 mAh g^−1^, respectively, corresponding to Coulombic efficiencies of 90.9% and 96.9%. The subsequent cycles still showed fast fading reversible capacities for EFID as same with previous case using LiPF_6_/EC‐DEC, while the subsequent cycles of FBND exhibited reversible capacities of ≈150 mAh g^−1^, which were similar to the previous case using LiPF_6_. This suggests that the two types of electrolytes did not exhibit significantly different performance in this case.

The long‐term cycling performances of EFID and FBND were examined for material stability at a current density of 0.2 A g^−1^, as shown in Figure 3d. FBND demonstrated a stable cycling performance, while EFID exhibited a fading capacity as observed before. After a decreasing trend up to the 50th cycle (activation cycles), the specific capacities of EFID and FBND remained at 95 and 40 mAh g^−1^, respectively. Even after 200 cycles, their capacities were maintained at around 85 and 25 mAh g^−1^, corresponding to capacity retentions of 89% and 62% compared to the capacity at the 50th cycle, respectively. Interestingly, the further cycling of FBND showed a capacity uplifting behavior with capacities of 88.1, 92.3, and 90.1 mAh g^−1^ at the 400th, 1600th, and 2000th cycle, respectively, corresponding to capacity retentions of 92.6%, 93.4% and 91.2% compared to the capacity at the 50th cycle (after activation cycles), which is mainly attributed to conventional activation process, i.e., the embedded activate material (AM) in cracking surface of AM particles could be exposure to further participate in the conversion reaction during the discharge/charge cycling process.^[^ ^46^ ^]^ Compared with the previously reported representative organic cathode materials after activation cycles, FBND showed relatively better cycling performance with high capacity retention as shown in Figure 3c.^[^ ^8^, ^11^, ^23^, ^31^, ^32^, ^33^, ^47^, ^48^, ^49^, ^50^, ^51^, ^52^, ^53^, ^54^, ^55^ ^]^ These results demonstrate that the long‐term cycling performance is crucial to consider the electrochemical stability and the solubility of the molecular structure of the active material.

**Figure 4:**
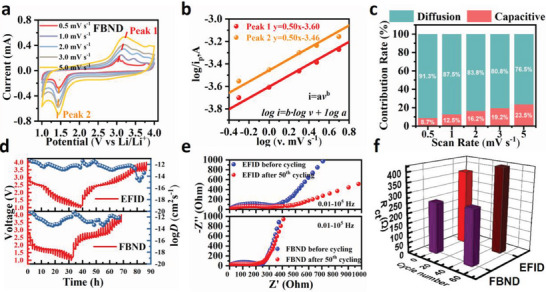
Electrode kinetics of EFID and FBND: a) CV curves of FBND at various scan rates; b) b‐values for FBND electrodes plotted as a function of potential for cathodic sweeps; c) Capacitive and diffusion contribution ratios at different scan rates; d) GITT curve in a half‐cell within the potential window of 1.0–4.0 V for EFID and FBND; e) Nyquist plots of EFID and FBND before and after the 50th cycle; f) Charge‐transfer resistance (*R*_ct_) of EFID and FBND electrodes before and after the 50th cycle.

To track the origin of the kinetics behavior of EFID and FBND cathodes for their poor rate performance, quantitative analyses were performed using cyclic voltammetry (CV) at various scan rates to separate pseudocapacitive and diffusion contributions, as shown in **Figure** **4**a and Figure S10a (Supporting Information). The current, i, as a function of the scan rate, v, was used to determine the b‐values by following the power‐law relationship *i*  = *av^b^* , where a and b are adjustable coefficients, with b‐values deduced from the slope of the linear fit between log i and log v. As all we know, when b‐value equal to 0.5, it represents diffusion‐controlled electrochemical process, which is indicative of a faradaic intercalation process.^[^ ^56^ ^]^ The peak one (oxidation peak) in Figure S10b (Supporting Information) and the two peaks (oxidation and reduction peak) in Figure 4b show the good linear fit between log i and log v when the b‐value was fixed as 0.5 with high R‐squared showed 0.98, 0.97 and 0.95 for the three fitting line, respectively, indicating they are diffusion‐controlled processes. However, the peak two (reduction peak) in Figure S10b (Supporting Information) for EFID showed bad linear fit with low R‐squared of 0.41 compared with its counterpart, the peak one (oxidation peak), indicating it is bad reversible electrochemical performance due to its good solubility.

As the scan rate increases from 0.5 to 5 mV s^−1^, the electrochemical behaviors are further described in the CV curves to quantitatively calculate the proportion of diffusion‐controlled capacitive contribution to the total capacity. The current response, i, can be expressed as a combination of surface capacitive‐dominated and diffusion‐controlled processes, as shown in Equation (1)^[^ ^56^ ^]^:

(1) $$i\;=k_{1}\;v+k_{2}v^{1/2}$$

Here, *k*_1_ *v* and *k*_2_ *v* ^1/2^ represent the capacitive process and embedded diffusion process, respectively. The calculated proportion of diffusion‐controlled capacitive contribution for EFID is estimated to be 80.4% of the total capacity at a scan rate of 0.5 mV s^−1^, as shown in Figure S10c (Supporting Information). The ratio of diffusion‐controlled capacitive contribution decreases from 80.4% to 55.6% with the increased current density, indicating that the capacitive contribution gradually becomes dominant under high scan rates. Similarly, the diffusion‐controlled capacitive contribution of FBND decreases from 91.3% to 76.5% at elevated scan rates from 0.5 to 5 mV s^−1^, indicating a diffusion‐controlled character at such high scan rates as shown in Figure 4c, which is consistent with the result as previous b‐value analysis. Thus, the rate performance of EFID and FBND were limited by the ion transport rate from electrolyte into the electrode.

Furthermore, the intermittent titration technique (GITT) is used to explore the ion‐diffusion kinetics and provide evidence for the poor rate performance of EFID and FBND. The average diffusion coefficient of Li+ ions ($D_{Li^{+}}$) in FBND is calculated to be 10^−13^–10^−11^ cm^2^ s^−1^ during the discharge and charge process, as shown in Figure 4d. This value is slightly higher than EFID's $D_{Li^{+}}$ (10^−15^–10^−11^ cm^2^ s^−1^), but both are similar to most other organic cathodes and lower than specific cases with good rate performance (e.g., PTN with a lower resistance of ≈50 Ω and high $D_{Li^{+}}$ on 10^−11^ cm^2^ s^−1^) as shown in Table S1 (Supporting Information), which confirms the poor rate ability of FBND. This issue will be addressed in future studies to increase the ion conductivity in the electrolyte and electrode.

The electrochemical impedance spectroscopy (EIS) measurement was simultaneously conducted in Figure 4e. FBND cathode exhibits a charge‐transfer resistance (*R*_ct_) of 220 Ω before cycling and 250 Ω after 50 cycles, while EFID shows a higher resistance, reaching 355 Ω before cycling and 405 Ω after cycling, as shown in Figure 4f. The essentially enhanced electron transport after making the isomerization from EFID to FBND is evident. However, the resistance of EFID and FBND is still larger than that of high‐rate organic materials,^[^ ^55^, ^57^ ^]^ indicating that this is another factor to be addressed in future studies to improve the rate ability of the active material.

**Figure 5:**
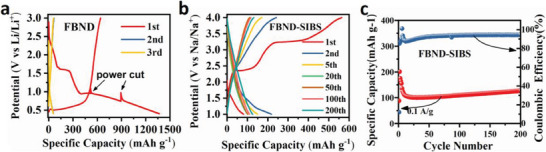
a) Discharge‐charge profiles of the FBND electrode with lithium foil as the counter electrode, tested at 0.1 A g^−1^ in the potential range of 0.3–3 V. b) Discharge‐charge profiles of the FBND electrode with sodium foil as the counter electrode, tested at 0.1 A g^−1^ in the potential range of 1–4 V. c) Cycling performance of the FBND electrode with sodium foil as the counter electrode.

As per previous reports, certain fused aromatic rings have demonstrated an exceptionally high storage capacity upon deep discharge, as observed with NTCDA^[^ ^29^ ^]^ for lithium battery anodes and PTCDA^[^ ^53^ ^]^ for sodium battery anodes. Hence, we investigated FBND in lithium batteries to understand whether and how many Li ions can be incorporated into it. The FBND electrode was discharged to 0.03 V instead of 1 V, revealing several potential stages, as shown in **Figure** **5**a.

The initial plateau at 1.6 V ends with approximately 200 mAh g^−1^ after a slope area from 3.0 to 1.6 V, contributing ≈100 mAh g^−1^, followed by another slope from 1.6 to 1.0 V, providing an additional 100 mAh g^−1^. These results are attributed to the formation of Li_2_FBND and the solid‐electrolyte interphase (SEI). The following plateau at 1.0 V concludes with ≈170 mAh g^−1^, indicating the incorporation of two more Li ions, forming Li_4_FBND, as proposed in Scheme S1 (Supporting Information). Subsequently, a long slope below 1.0 to 0.3 V delivers a capacity of ≈1100 mAh g^−1^, along with the formation of the Li_16_FBND structure as depicted in Scheme S1 (Supporting Information). This capacity and mechanism are reminiscent of Li_18_NTCDA,^[^ ^29^ ^]^ K_11_PTCDA,^[^ ^58^ ^]^ and Na_15_PTCDA.^[^ ^53^ ^]^

However, during the subsequent charge of Li_18_FBND, only a small reversible capacity of ≈650 mAh g^−1^ is achieved, corresponding to a low Coulombic efficiency of ≈40% in the potential range from 3 to 0.03 V, indicating that not all the lithium ions incorporated into the fused aromatic rings can be removed. This behavior is similar to that observed in the cases of NCTDA and PTCDA, as reported in previous studies.^[^ ^29^, ^53^ ^]^ The charge capacity rapidly decreases to 55 mAh g^−1^ in further cycling, indicating that pristine FBND may not be suitable as an anode material in lithium‐ion batteries. Nevertheless, the high capacity of FBND may present potential for primary lithium batteries.

Furthermore, we evaluated the electrochemical behavior of FBND in sodium batteries using coin cells with sodium foil as the anode electrode, glass fiber membrane as the separator, and 1.0 M NaPF_6_ in EC: DEC solution mixed with 1:1 volume ratio as the electrolyte. The FBND cathode also exhibited superior electrochemical performance in sodium batteries, delivering a higher first‐cycle sodium extraction capacity of 200 mAh g^−1^, as shown in Figure 5b. Interestingly, the initial discharge capacity is lower than initial charge capacity, indicating FBND show a P‐type organic cathode behavior^[^ ^59^, ^60^ ^]^ in sodium battery. After 200 cycles, a large capacity of 110 mAh g^−1^, comparable to that of lithium batteries, was observed for the FBND cathode, as shown in Figure 5c. This indicates that FBND possesses adaptability for multiple alkali‐ion energy storage. Further investigation into the detailed electrochemical performance and mechanisms in sodium batteries will be conducted in future studies.

### 2.3 Mechanism in Energy Storage for FBND

**Figure 6:**
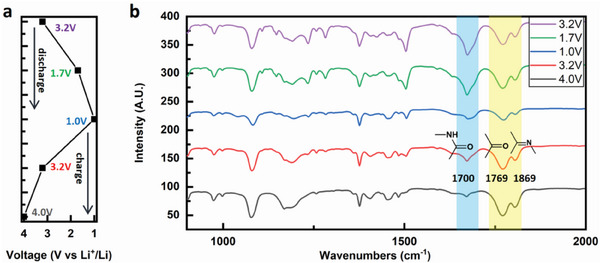
a) Representative discharge and charge profiles of FBND at a current density of 0.1 A g^−1^. b) Ex situ FTIR measurements at marked points between 1.0 and 4.0 V.

**Figure 7:**
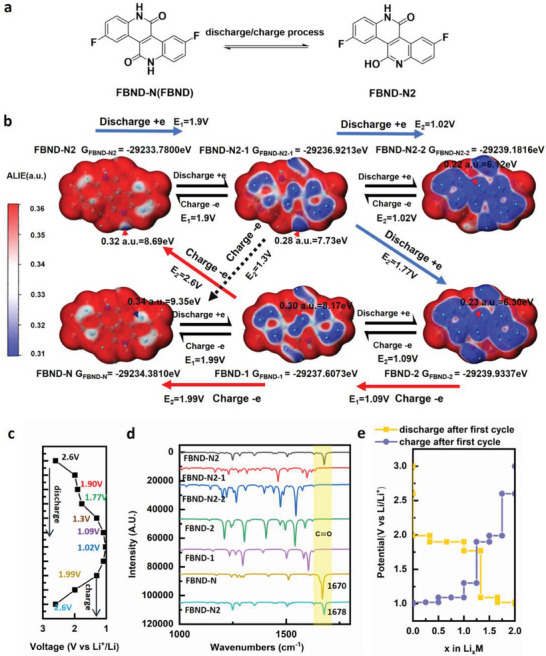
Electrochemical mechanism and DFT calculation result of FBND. a) The isomer of FBND during the discharge–charge process. b) Calculated Gibbs free energy (marked G) of FBND (FBND‐N represents FBND neutral state) and FBND‐N2, along with their reduction products, including radical ions (FBND‐1, spin‐up; FBND‐N2‐1, spin‐up) and divalent ions (FBND‐2, FBND‐N2‐2). c) The corresponding potential versus Li+/Li at each possible state. d) The corresponding infrared spectroscopy (IR) at each possible state. e) Discharge–charge profile of possible ways estimated by the calculated working voltage of each state.

The discharge‐charge profile of FBND at a current density of 0.1 A g^−1^ is illustrated in **Figure** **6**a. To further investigate the discharging and charging processes, ex situ FTIR spectroscopy was employed, and the results are presented in Figure 6b. Two regions in Figure 6b are of particular interest: the light blue region corresponds to the stretching vibration of the C═O bond of imide groups at 1700 cm^−1^, consistent with the FTIR spectroscopy of FBND powder as shown in Figure 1a. However, the yellow region exhibits new peaks at 1769 and 1869 cm^−1^, different from FBND's FTIR spectroscopy, which are typically associated with vibrations of the C═O bond or C═X (X═C, N, S, etc.). This observation suggests that the chemical structure of FBND may have undergone changes, such as transitioning from FBND‐N to FBND‐N2 state due to the isomerization of the imide group as shown in **Figure** **7**a. This finding aligns with the changing trending in the simulated IR spectrum results of FBND‐N and FBND‐N2, where the C═O bond signal red‐shifts from 1670 cm^−1^ in FBND‐N to 1678 cm^−1^ in FBND‐N2, as depicted in Figure 7d. Both regions with new peaks are considered active redox centers.

During the discharging process, the signals of the C═O bond of FBND at 1700 cm^−1^, the C═O at 1769 cm^−1^, and C═N bond at 1869 cm^−1^ in FBND become weak from 3.2 to 1.0 V and almost disappear after discharging to 1.0 V. This indicates that in the discharging process, the C═O and C═N bonds of FBND and FBND‐N2 are successively lithiated. In the charging process, the signal of the yellow region gradually emerges, suggesting the reverse of the discharge process for the C═O and C═N bonds of FBND‐N2. However, the C═O bond signal of FBND at 1700 cm^−1^ does not recover, indicating that the reduction state of FBND is more likely to change to FBND‐N2 after charging beyond 2.6 V, corresponding to the plateau in the charge process as observed in Figure 2b. This observation is consistent with the separate couples of redox peaks as shown in Figure 4a.

To further investigate the electrochemical redox mechanism during the discharge‐charge process, we employed Density Functional Theory (DFT) calculations to study the FBND and FBND‐N2 states, with their neutral states marked as FBND‐N and FBND‐N2, respectively. The FBND and FBND‐N2 states accepting one electron were denoted as FBND‐1 and FBND‐N2‐1, while the states accepting two electrons were denoted as FBND‐2 and FBND‐N2‐2.

To predict the reduction process of FBND‐N and FBND‐N2 during the discharge/charge process, we calculated the Average Local Ionization Energy (ALIE)^[^ ^61^ ^]^ which reveals the positions on the surface that are preferred sites for electrophile (e.g., lithium cation, Li+). These positions indicate where electrons are easily ionized, representing the highest occupied molecular orbit area on the molecule. The geometrical optimizations and frequency calculations at the B3LYP/6‐311+(d,p) level were performed using Gaussian 09, and ALIE analysis was carried out using the Multiwfn software.^[^ ^62^ ^]^ We found that FBND‐1 and FBND‐N2‐1 have surface minima positions with energies of 8.17 and 7.73 eV as shown in Figure 2b, respectively, located near the carbon position of the C═O bond in FBND‐1 and the nitrogen position of the C═N bond in FBND‐N2‐1. These positions correspond to discharging to 1.9 and 1.99 V versus Li+/Li, respectively, which aligns with the Equation (2) in the DFT calculation section of the experimental section, and the simulated IR spectrum of FBND.

Similarly, FBND‐2 and FBND‐N2‐2 have surface minima energies of 6.30 and 6.12 eV, respectively, located near the carbon atom of the C═O bond in the imide structure. The corresponding working voltages versus Li+/Li are 1.09 and 1.02 V. As per the experimental ex situ FTIR data in Figure 6b, FBND tends to transform into FBND‐N2. Consequently, FBND‐1 could be charged to FBND‐N2 or discharged to FBND‐N2‐2, while FBND‐N2‐1 could be charged to FBND‐N or discharged to FBND‐2, with corresponding working voltages of 2.6, 0.3, 1.3, and 1.77 V, respectively. The 0.3 V working voltage could be disregarded as it lies beyond the experimental voltage range. These possible working voltages in the range of 1–3 V are summarized in Figure 7c.

To simulate the experimental discharge and charge processes of FBND, we considered three possible discharge processes (**labeled 1‐3**) and four possible charge processes (**labeled 4‐7**) as shown in Figure S11 (Supporting Information). Notably, the discharge/charge curves in experimental data, as shown in Figure 3b, exhibit slope regions before and after the plateau. These features suggest the presence of multiple‐step redox reactions. To approximate the experimental discharge and charge processes of FBND, we abstracted parts of the discharge process **1‐3** in average to merge the discharge process represented by the yellow line in Figure S11a (Supporting Information). Similarly, we abstracted parts of the discharge process **4‐7** in average to construct the charge process represented by the violet line in Figure S11b (Supporting Information). These abstracted processes were then combined into a single figure, as depicted in Figure 7e. Notably, the proposed discharge and charge processes in Figure 7e closely mirror the experimental discharge and charge processes in Figure 3b, thus confirming that the electrochemical redox process is composed of these seven pathways, as illustrated in Figure S11 (Supporting Information), between the FBND‐2 and FBND‐N2 states.

## 3 Conclusion

In summary, this study successfully synthesized a novel insoluble FBND organic cathode through the isomerization of an isoindigo derivative, EFID, for use in high‐performance LIBs. By virtue of its imide structure and high π‐conjugated system, FBND effectively addresses the challenge of solubility that is often associated with organic cathode materials in organic electrolytes. Exploiting the limited solubility of FBND in organic electrolytes led to the manifestation of notable electrochemical characteristics, encompassing a high capacity of 175 mAh g^−1^ at a current density of 0.05 A g^−1^, coupled with a commendable rate capacity of 90 mAh g^−1^ at 0.2 A g^−1^, thereby demonstrating commendable cycling stability over 2000 cycles, accompanied by an excellent retention rate of 90%. Simultaneously, this study delved into the potential utility of FBND as both an anode in LIBs and a cathode in SIBs. The initial cycle discharge capacity of FBND as an anode registered an impressive 1500 mAh g^−1^ when discharging to 0.3 from 3 V. Nonetheless, subsequent cycling experiments revealed a diminished reversible capacity. Similarly, in the context of SIBs, FBND exhibited a commendable cathode capacity of 110 mAh g^−1^ at a current density of 0.1 A g^−1^ across 200 cycles. The employment of ex FIRT measurements and DFT calculations validated the multiple‐step electrochemical redox mechanism of the discharge/charge process of the FBND cathode, thereby affirming its viability for deployment in stable energy storage systems. In essence, this study proposes a straightforward and facile strategy to ameliorate the dissolution challenge associated with organic electrodes through the implementation of the FBND framework subsequent to EFID isomerization. This framework holds the potential to serve as a favorable molecular scaffold, facilitating the advancement of cycling stability and achieving elevated capacity by the introduction of imine or carbonyl groups into the framework.

## 4 Experimental Section

### Material Synthesis

*(E)−5,5′‐difluoro‐[3,3′‐biindolinylidene]−2,2′‐dione (EFID)*: In a 100 ml round‐bottomed two‐necked flask equipped with a reflux condenser and magnetic bar, 3.0 g (18.18 mmol) of 5‐fluoroindoline‐2,3‐dione and 3.0 g (19.86 mmol) of 5‐fluoroindolin‐2‐one were mixed with 50 ml of acetic acid. Then, 0.5 ml of concentrated hydrochloric acid (HCl) was added dropwise, and the mixture was heated to 105 °C. The color of the system changed from bright red to dark red within 1 h. The temperature was maintained at 105 °C for 6 h and then cooled down to room temperature. The resulting dark red precipitate was filtered, washed several times with water and ethanol, and finally dried under vacuum, yielding ≈5.2 g (95%) of the dark red product as a step in Scheme S1 (Supporting Information). No further purification was performed for the subsequent reaction. The proton nuclear magnetic resonance spectrum (δH) (400 MHz; DMSO with Me_4_Si) showed peaks at 11.0 ppm (2H, s, CONH), 9.0 ppm (2H, m, Ph), 7.25 ppm (4H, m, Ph), and 6.85 ppm (4H, m, Ph). The high‐resolution mass spectrometry (HRMS) (LC‐MS) gave a mass‐to‐charge ratio (m/z) of [M]+ calcd for C_16_H_8_F_2_N_2_O_2_+H as 299.0625.

*3,9‐difluoro‐ 4b,6,10b,12‐ tetrahydrodibenzo [c,h][2,6] naphthyridine −5,11‐dione (key intermediate)*: In a 100 ml round‐bottomed two‐necked flask with a magnetic bar, 3.0 g (10.07 mmol) of EFID and 1.9 g (29.69 mmol) of zinc powder were combined with 50 ml of tetrahydrofuran (THF). Then, 3.0 g (26 mmol) of trifluoroacetic acid (TFA) was added, and the mixture was stirred at room temperature for 10 h. The dark red suspension turned into a white‐grey suspension. The stirring was continued for an additional 2 h, and then 30 ml of concentrated hydrochloric acid (HCl) was carefully added. The mixture was stirred for 2 more hours to ensure complete reaction of the remaining zinc powder. The zinc powder was thoroughly cleaned before the next reaction. After most of the zinc powder reacted with concentrated HCl, the mixture was poured into 100 ml of water, and the resulting white precipitate was filtered and dried under vacuum, yielding ≈2.8 g of the product.

Next, 2.8 g (9.3 mmol) of the above product was added to a 100 ml round‐bottomed two‐necked flask equipped with a reflux condenser and magnetic bar. To this, 20 ml of concentrated HCl and 20 ml of dioxane were added, and the mixture was heated to reflux for 18 h until the starting material was completely reacted. The mixture was then cooled to room temperature, and the white or light‐yellow powder was filtered, washed with water and ethanol, and dried under vacuum, yielding the key intermediate of 2.3 g (82%). Only the proton magnetic resonance spectrum (H‐NMR) was measured for this product since it was poorly soluble in common solvents. The H‐NMR spectrum showed peaks at 10.57 ppm (2H, s, CONH), 7.19–7.18 ppm (4H, m, Ph), 6.95 ppm (2H, s, Ph), and 4.23 ppm (2H, s, COCH). The mass spectrometry (MS) (GC‐MS) gave a mass‐to‐charge ratio (m/z) of [M]+ calcd for C_16_H_10_F_2_N_2_O_2_ as 300.0.

*3,9‐difluoro‐6,12‐dihydrodibenzo[c,h][2,6]naphthyridine‐5,11‐dione (FBND)*: To a solution of the key intermediate 2.0 g (6.7 mmol) in N,*N*‐dimethylformamide (DMF), 3.78 g of potassium carbonate (K_2_CO_3_) (4 eq, 26.8 mmol) was added. The resulting suspension was stirred for 48 h under ambient air. The yellow solid FBND (1.97 g) obtained was filtered, washed with water, then with tetrahydrofuran (THF) and ethanol, and finally dried under reduced pressure with yield of ≈99%. FBND was found to be insoluble in common solvents, and its structure was confirmed using infrared spectroscopy (FT‐IR) as shown in Figure 1a.

### Materials Characterizations

X‐ray diffraction (XRD) analysis was performed on a D8 Advance X‐ray diffractometer (Bruker AXS, Germany) with Cu Kα radiation in the range of 2θ = 5‐80°. The microscopic topography of the samples was characterized by scanning electron microscopy (SEM) using a Nano SEM 200 instrument (FEI, United States). Transmission electron microscopy (TEM) and high‐resolution transmission electron microscopy (HRTEM) along with high‐angle annular dark‐field scanning transmission electron microscopy (HAADF‐STEM) were used to record the morphology, internal microstructure, and elemental mapping of the samples. Ex‐situ Fourier‐transform infrared spectroscopy (FT‐IR) was recorded using a PerkinElmer Frontier FT‐IR instrument. Proton nuclear magnetic resonance (H‐NMR) spectra were acquired using a zhongkeniujin WNMR‐I 400 MHz spectrometer with dimethyl sulfoxide‐d6 (DMSO‐d6) containing tetramethylsilane (TMS, 0.03%) as the solvent.

### Electrochemical Characterizations

All electrochemical tests were performed using CR2032 coin cells. The working electrodes were prepared by mixing the organic electrode material with Ketjen Black and polyvinylidene difluoride (PVDF) in a weight ratio of 7:2:1. The mixture was then dispersed in N‐methyl‐2‐pyrrolidone (NMP) and coated onto an aluminum (Al) foil using the scraper method to prepare the slurry. After drying overnight under vacuum at 100 °C, a disc with a diameter of 12 mm was cut as the working electrodes. The loading mass of the electrodes was 1 (±0.1) mg cm^−2^ based on active materials. Lithium foils and Celgard 2500 were used as the counter electrode and separator, respectively. A 1 M LiPF_6_ in ethylene carbonate/diethyl carbonate (EC/DEC) solution mixed at a ratio of 1:1 by volume and a 1 M LiTFSI in 1,3‐dioxolane/dimethoxymethane (DOL/DME) solution mixed at a ratio of 1:1 by volume were used as the electrolyte. The coin cells were assembled in an argon‐filled glovebox with H_2_O and O_2_ levels of <0.01 ppm.

### Electrochemical Test

The electrochemical performance of EFID and FBND was evaluated by galvanostatic charge/discharge (GCD) using a NEWARE CT‐4008Tn instrument, cyclic voltammetry (CV) curves, and Electrochemical Impedance Spectroscopy (EIS) using a CHI 660E electrochemical workstation. The measurements were carried out within a potential range of 1.0 – 4.0 V and 1.5 – 3.8 V and a frequency range of 0.01–10^5^ Hz, respectively.

### DFT Calculation

Gaussian 09 software was used to perform the geometrical optimizations and frequency calculations at the B3LYP/6‐311+ (d,p) level. The solvation effect of the electrolyte was considered using the IEFPCM solvation model, with acetonitrile (ε = 35.7) as the solvent, approximating the dielectric constant of the electrolyte (EC/DEC mixture in a ratio of 1:1 by volume) used in this work. The HOMO/LUMO drawing and average local ionization energy (ALIE) analysis were performed using Mutliwfn software.^[^ ^62^ ^]^ The reduction potential of n‐type molecules (from P(x) to P(x+1)) in solution was computed using Equation (2),^[^ ^63^ ^]^

(2) $$E_{x\;}\;\left(\left(Li+|Li\right)\right)=\;-\frac{\Delta G}{nF}-1.24V\;=\;-\frac{\left(G_{P\left(x+1\right)}-G_{P\left(x\right)}\right)}{F}-1.24V$$

where *E*_*x*_((*Li* + |*Li*)) is the reduction potential (V versus Li+/Li) for the x‐th (x = 1 or 2) step, G is the Gibbs free energy (eV) of the specified molecule in solution, ΔG is the Gibbs free energy difference (eV) during the reduction process in solution, and F is Faraday's constant (C/mol). The subtraction of 1.24 V was used to convert the absolute reduction potential into the potential versus Li+/Li.

### Theoretical Specific Capacity

The theoretical specific capacity of FBND and EFID was calculated using Equation (3),^[^ ^26^ ^]^

(3) $$C_{theor}=\frac{nF}{3.6\times M}\;$$

where n, F, and M are the number of accepted electrons (*n* = 2), Faraday's constant (C mol^−1^), and the molecular weight of EFID and FBND, respectively.

## Conflict of Interest

The authors declare no conflict of interest.

## Supporting information

Supporting Information

## Data Availability

The data that support the findings of this study are available from the corresponding author upon reasonable request.
