# Supplementary material for: Long Cycle Life for Rechargeable Lithium Battery using Organic Small Molecule Dihydrodibenzo[c,h][2,6]naphthyridine‐5,11‐dione as a Cathode after Isoindigo Pigment Isomerization
Source: Adv Sci (Weinh). 2023 Nov 30;11(4):2307134. doi: 10.1002/advs.202307134 (PMC10811468; doi:10.1002/advs.202307134)
Supplement: Supplementary file 1 — Supporting Information [file ADVS-11-2307134-s001.pdf]

ADVANCED SCIENCE
Open Access

Supporting Information

for *Adv. Sci.*, DOI 10.1002/advs.202307134

Long Cycle Life for Rechargeable Lithium Battery using Organic Small Molecule Dihydrodibenzo[c,h][2,6]naphthyridine-5,11-dione as a Cathode after Isoindigo Pigment Isomerization

Mingcong Yang, Wei Hu, Jun Li, Tao Chen, Shiqiang Zhao, Xi'an Chen, Shun Wang and Huile Jin**

Long Cycle Life for Rechargeable Lithium Battery using Organic Small Molecule Dihydrodibenzo[c,h][2,6]naphthyridine-5,11-dione as Cathode after Isoindigo Pigment Isomerization

Mingcong Yang, Wei Hu, Jun Li, Tao Chen, Shiqiang Zhao, Xi an Chen, Shun Wang, Huile Jin**

M. Yang, W. Hu, J. Li, S. Zhao, X. Chen, S. Wang, H. Jin

Key Lab of Advanced Energy Storage and Conversion, Zhejiang Province Key Lab of Leather Engineering, College of Chemistry and Materials Engineering, Wenzhou University Wenzhou, Zhejiang 325035, China

E-mail: junli@wzu.edu.cn; huilejin@wzu.edu.cn

M. Yang, H. Jin

Zhejiang Engineering Research Center for Electrochemical Energy Materials and Devices, Institute of New Materials and Industrial Technologies, Wenzhou University Wenzhou, Zhejiang 325035, China

M. Yang, T. Chen

Department of Materials Science and Engineering, School of Chemistry and Materials Science, University of Science and Technology of China, Hefei, Anhui Province 230026, China

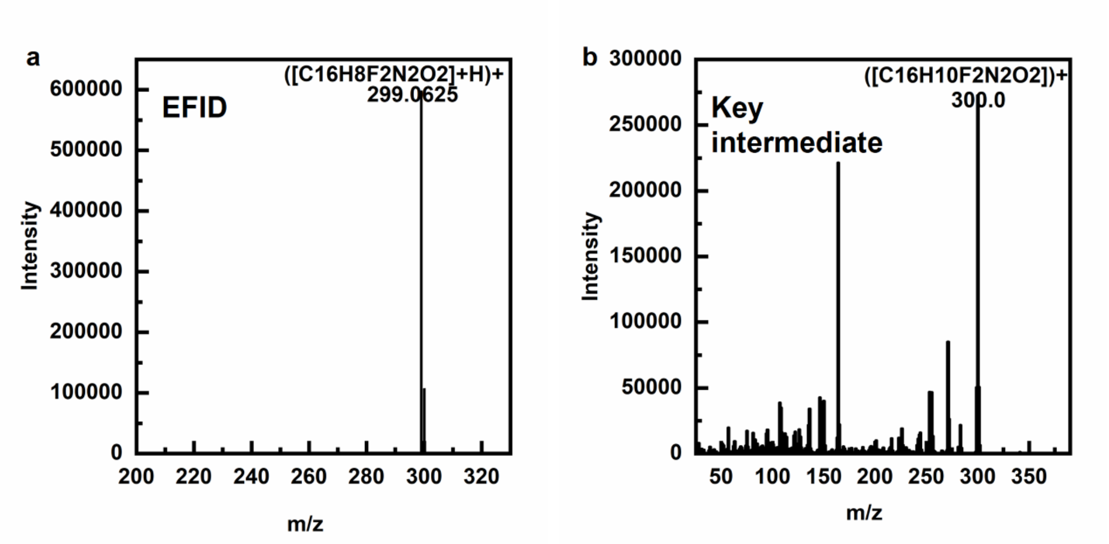

SI Figure 1. structure characterization of EFID and key intermediate by Mass Spectra as (a) and (b), respectively.

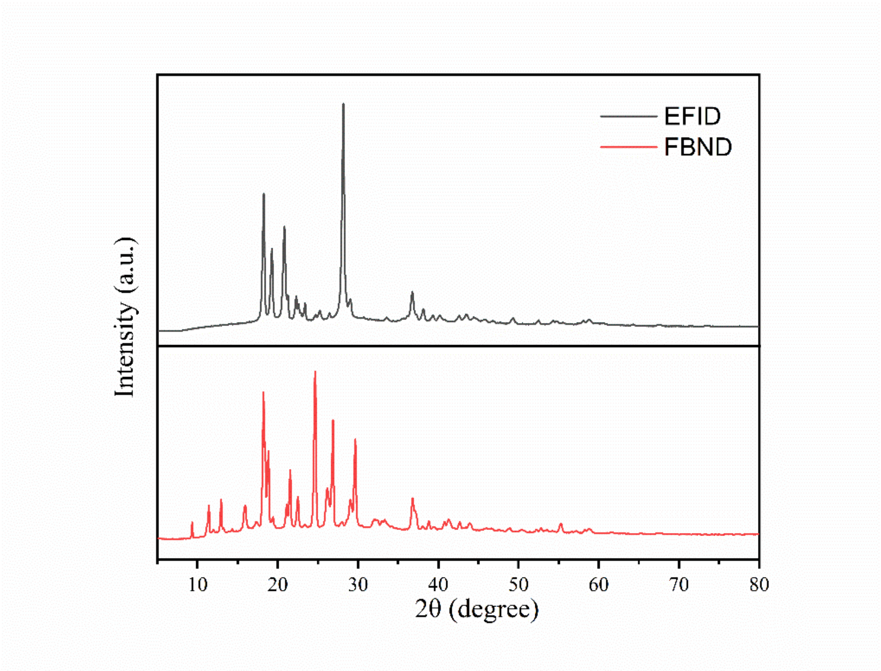

SI Figure 2. structure characterization of EFID and FBND by PXRD pattern as black and red curve, respectively.

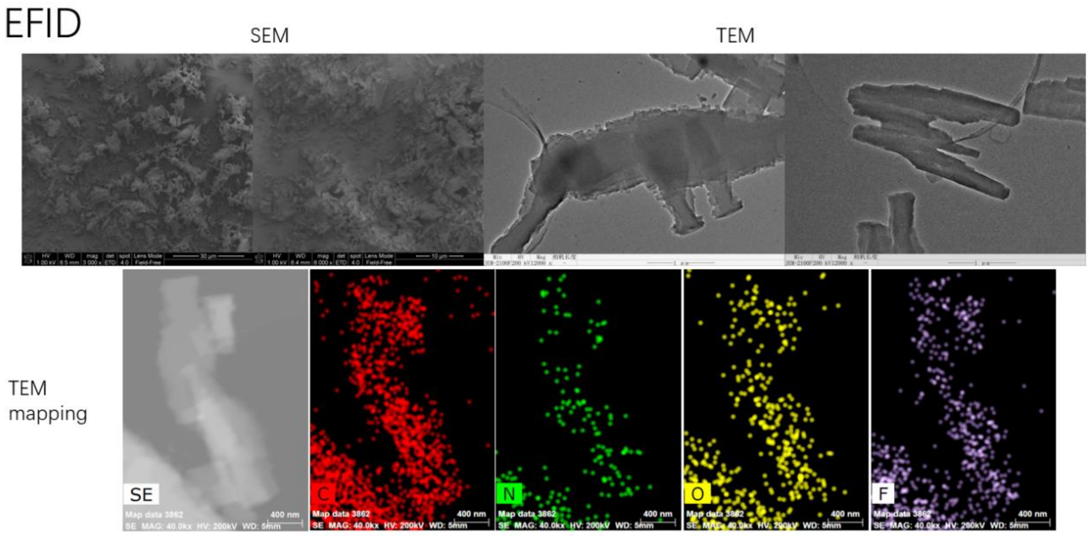

SI Figure 3. Structure and morphological analysis of EFID with SEM, TEM and the corresponding EDS mapping images for C, N, O and F of EFID

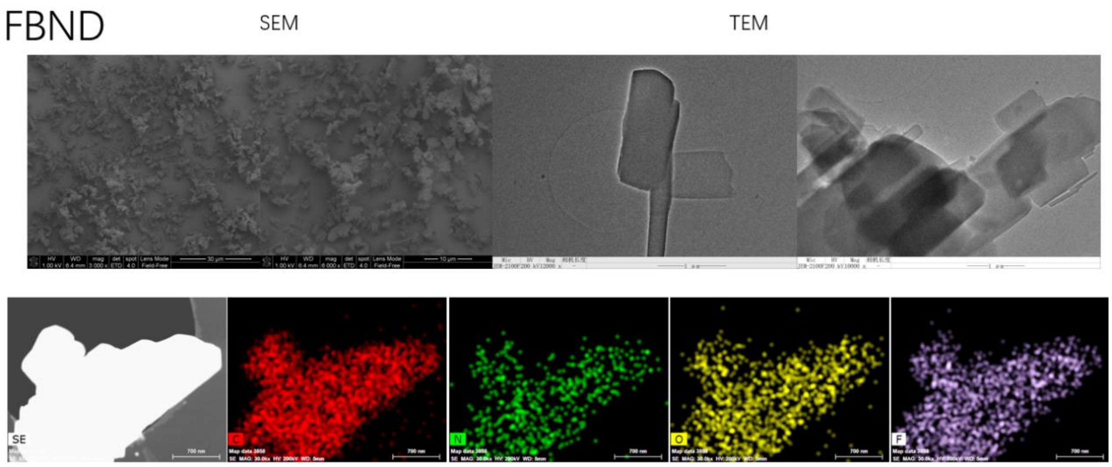

SI Figure 4. Structure and morphological analysis of FBND with SEM, TEM and the corresponding EDS mapping images for C, N, O and F of FBND

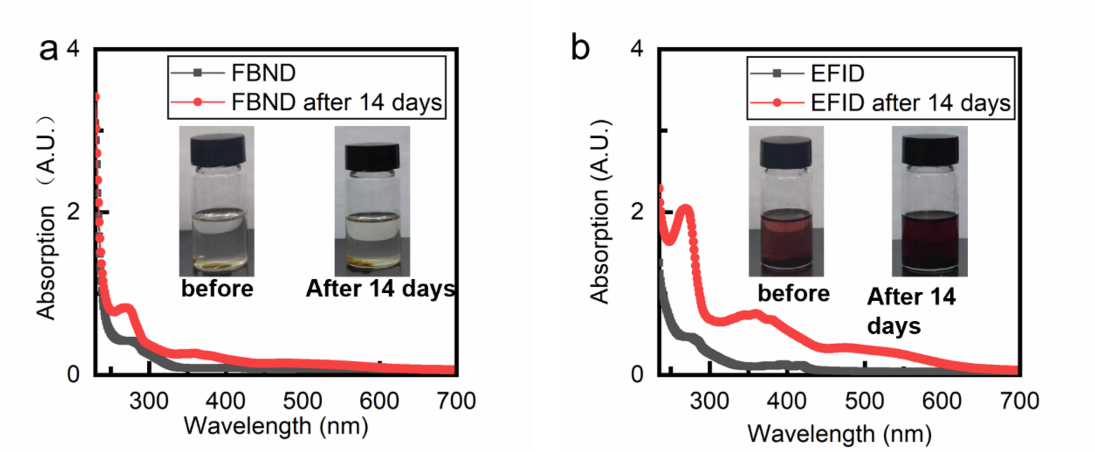

SI Figure 5 Dissolution test for FBND and EFID. a) UV/ vis spectra of FBND powder before and after 14 days. b) UV/vis spectra of EFID powder before and after 14 days

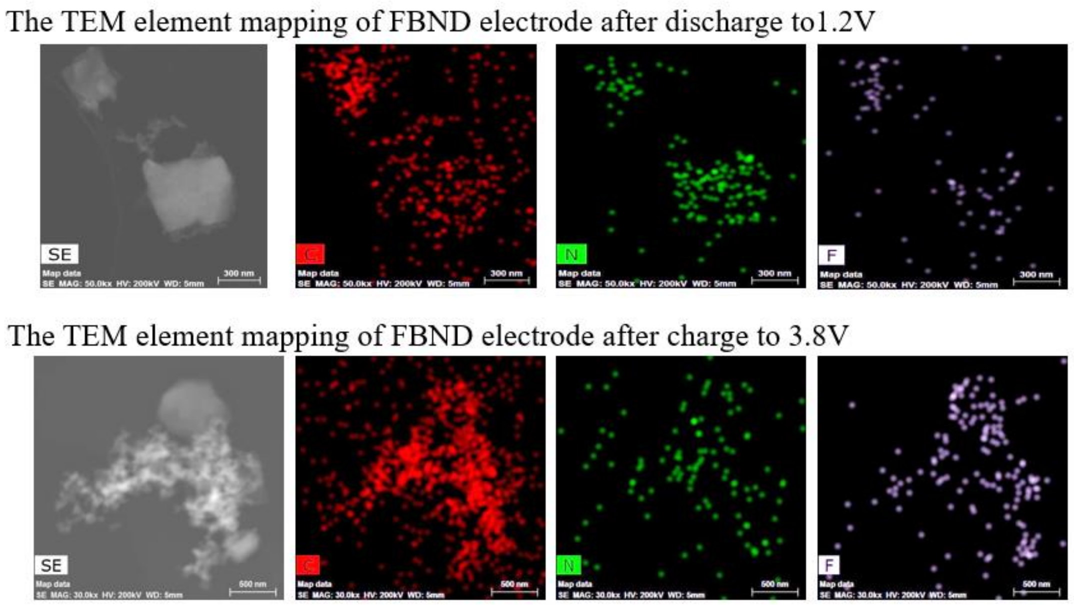

SI Figure 6 Dissolution test for FBND electrode. EDS mapping of FBND electrode after discharge to 1.2V and after charge to 3.8V.

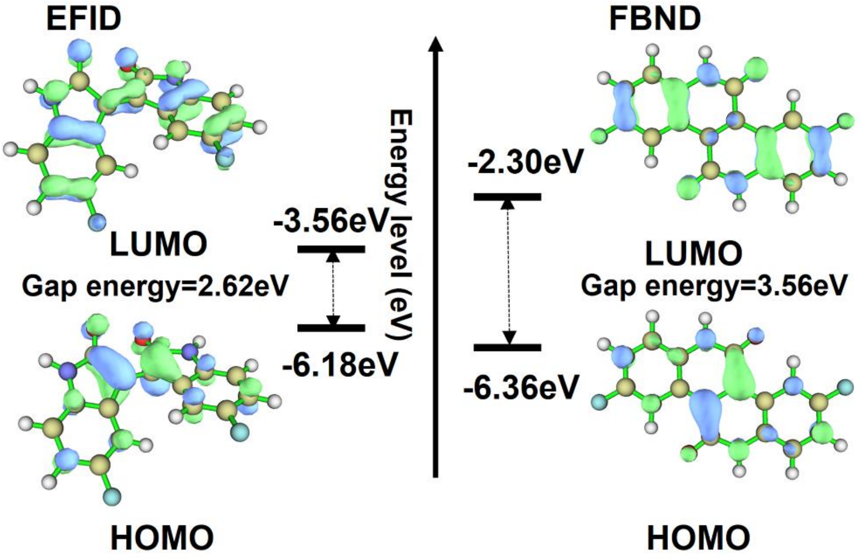

SI Figure 7 Molecular structures and HOMO/LUMO energy levels of EFID and FBND calculated by Gaussian 09 at B3LYP/6-311+(d,p) level and generated by the Multiwfn software

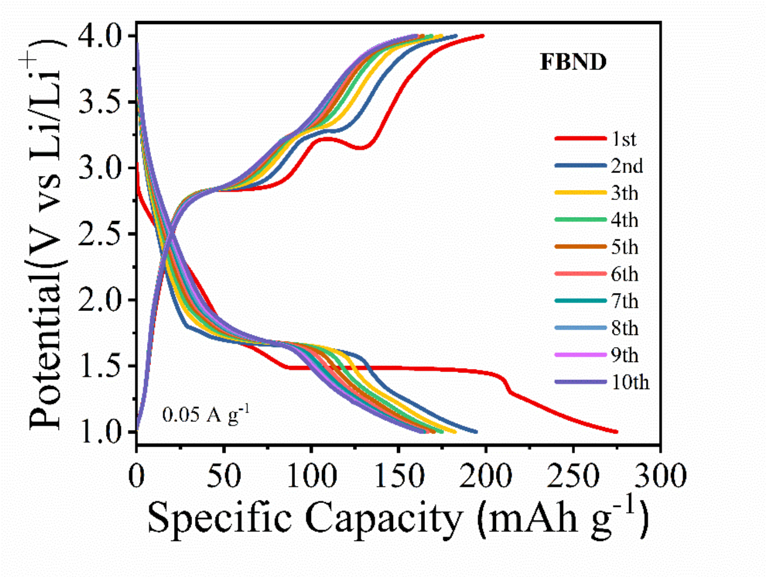

SI Figure 8 Electrochemical performance of FBND with LiPF6/EC-DEC (volume ratio of 1:1) as electrolyte: Discharge-charge curves of FBND at the current density of 0.05A/g

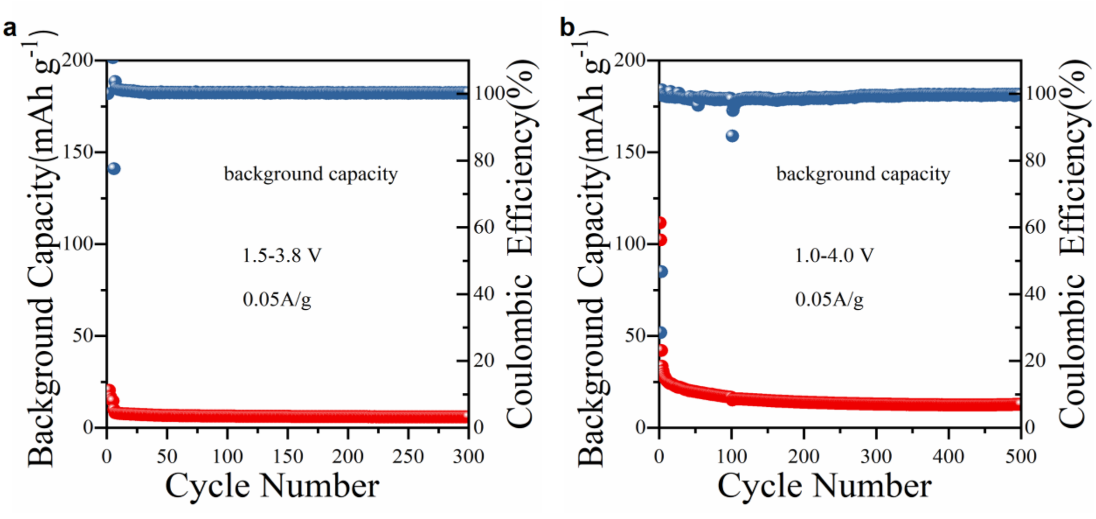

SI Figure 9 Electrochemical performance of the conductive material of KB and GO mixture with LiPF6/EC-DEC (volume ratio of 1:1) as electrolyte: (a,b) Discharge-charge capacity at the current density of 0.05A/g;

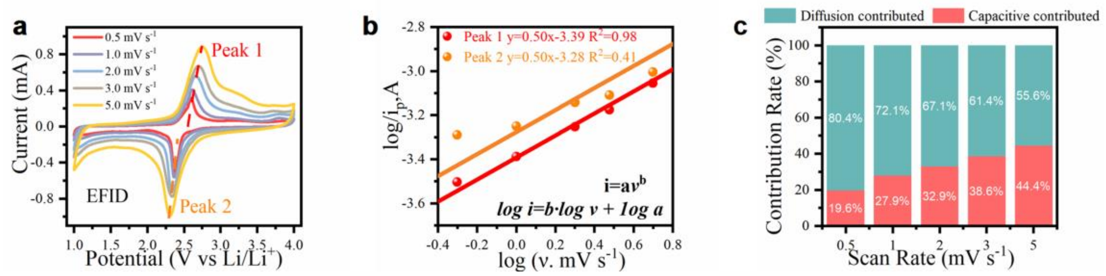

SI Figure 10 Electrode kinetics of EFID: (a) CV curves of EFID at various scan rates; (b) b-values for EFID electrodes plotted as a function of potential for cathodic sweeps; (c) Capacitive and diffusion contribution ratios at different scan rates;

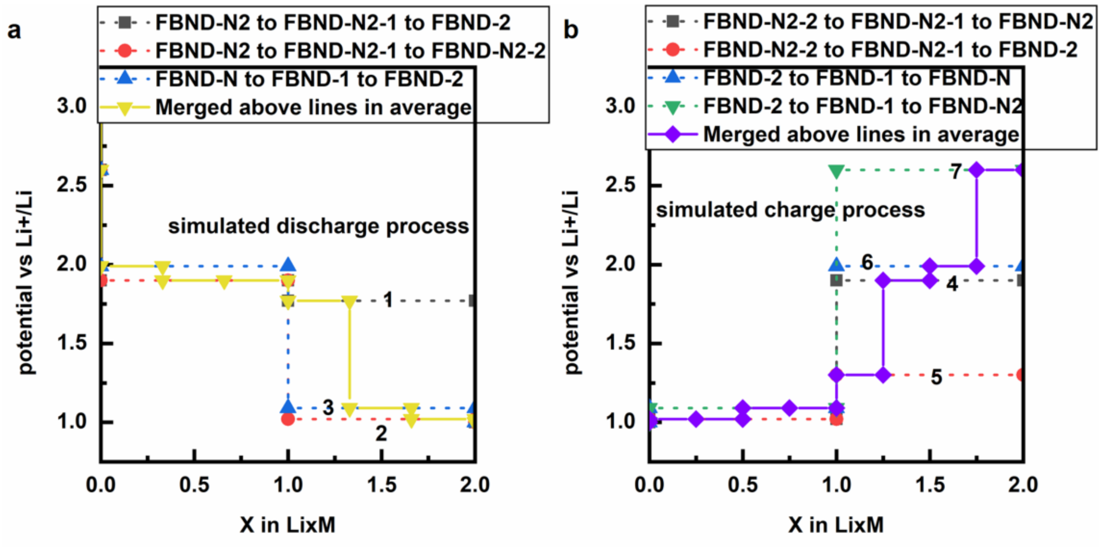

SI Figure 11 The simulated possible ways for (a) discharge and (b) charge process of FBND.

SI Scheme 1. Schematic diagrams for the proposed electrochemical reactions of FBND with lithium foil as counter in potential range of 3-0.3V

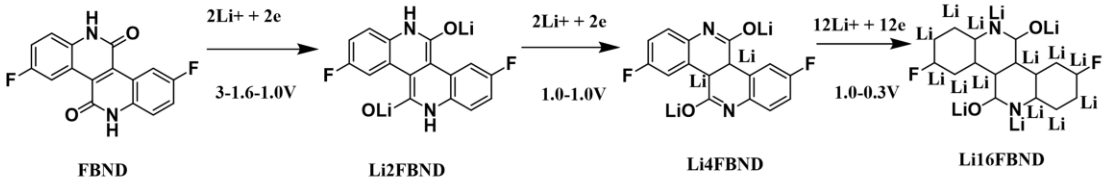

SI Table 1. Comparison of ionic diffusivities of Fe-HATNTA with reported organic and inorganic cathodes

Electrode material	diffusivities ($cm^2\ s^{-1}$)	Ref.
**FBND**	10^{-13}–10^{-11}	This work
**EFID**	10^{-15}–10^{-11}	This work
**Fe-HATNTA（MOF）**[1]	~10^{-9}	1
**PTCDA/RGO/CNT**[2]	1.83×10^{-12}	2
**PTN**[3]	~10^{-9}	3
**PBQDS**[4]	6×10^{-13}	4
**NTAQ**[5]	7.92×10^{-14}	5
**PAQS@Mxene**[6]	8.0×10^{-8}	6
**PMAQ**[5]	6.85×10^{-16}	5
**(Co, Mn)$_2$O$_4$**[7]	1.8×10^{-15}	7
**Fe-doped LiMnPO$_4$@C**[8]	3.31×10^{-12}	8
**LiFePO$_4$** [9]	10^{-10}–10^{-16}	9

References

1. Wang, Y. *et al.* High-Rate Organic Cathode Constructed by Iron-Hexaazatrinaphthalene Tricarboxylic Acid Coordination Polymer for Li-Ion Batteries. *Adv. Sci.* **9**, 2205069 (2022).
2. Zhou, G. *et al.* Bioinspired Micro/Nanofluidic Ion Transport Channels for Organic Cathodes in High-Rate and Ultrastable Lithium/Sodium-Ion Batteries. *Adv. Funct. Mater.* **28**, 1804629 (2018).
3. Wang, J. *et al.* Conjugated diketone-linked polyimide cathode material for organic lithium-ion batteries. *Chem. Eng. J.* **444**, 136598 (2022).
4. Tran, N. A., Leprêtre, J. C. & Alloin, F. Poly(benzoquinonyldisulfide) as organic positve electrode for Mg and Li batteries. *Electrochim. Acta* **375**, 137990 (2021).
5. Ba, Z. *et al.* Benzoquinone-Based Polyimide Derivatives as High-Capacity and Stable Organic Cathodes for Lithium-Ion Batteries. *ACS Appl. Mater. Interfaces*

12, 807–817 (2020).
6. Gao, Y. *et al.* Interfacial Self-assembly of Organics/MXene Hybrid Cathodes Toward High-Rate-Performance Sodium Ion Batteries. *ACS Appl. Mater. Interfaces* **14**, 8036–8047 (2022).
7. Wu, B. *et al.* Construction of unique heterogeneous cobalt–manganese oxide porous microspheres for the assembly of long-cycle and high-rate lithium ion battery anodes. *J. Mater. Chem. A* **7**, 6149–6160 (2019).
8. Yang, H., Fu, C., Sun, Y., Wang, L. & Liu, T. Fe-doped LiMnPO4@C nanofibers with high Li-ion diffusion coefficient. *Carbon N. Y.* **158**, 102–109 (2020).
9. Churikov, A. V. *et al.* Determination of lithium diffusion coefficient in LiFePO4 electrode by galvanostatic and potentiostatic intermittent titration techniques. *Electrochim. Acta* **55**, 2939–2950 (2010).
